# Correlation of the serum cell division cycle 42 with CD4^+^ T cell subsets and in-hospital mortality in Stanford type B aortic dissection patients

**DOI:** 10.3389/fcvm.2024.1324345

**Published:** 2024-02-27

**Authors:** Hui Peng, Xugang Wang, Longfei Zhang, Yang Su, Jieli Yan, Xin Wu

**Affiliations:** ^1^ Department of Cardiac Surgery, Xingtai People’s Hospital, Xingtai, Hebei, China; ^2^ Department of Cardiovascular Surgery, The First Hospital of Hebei Medical University, Shijiazhuang, China; ^3^ Department of Cerebrovascular Neurosurgery, Xingtai People’s Hospital, Xingtai, Hebei, China

**Keywords:** cell division cycle 42, Stanford type B aortic dissection, CD4^+^ *T* cell subsets, in-hospital mortality, prognostic value

## Abstract

**Objective:**

Cell division cycle 42 (CDC42) regulates CD4^+^ *T*-cell differentiation and participates in vascular stiffness and atherosclerosis and is involved in the progression of Stanford type B aortic dissection (TBAD). This study aimed to explore the correlation between serum CDC42 level and CD4^+^ *T* cell subsets and in-hospital mortality in TBAD patients.

**Methods:**

Serum CDC42 and peripheral blood T-helper (Th) 1, Th2, and Th17 cells were detected in 127 TBAD patients by enzyme-linked immunosorbent assay and flow cytometry, respectively. Serum CDC42 was also quantified in 30 healthy controls.

**Results:**

Serum CDC42 was decreased in TBAD patients vs. healthy controls (median [interquartile range (IQR)]: 418.0 (228.0–761.0) pg/ml vs. 992.0 (716.3–1,445.8) pg/ml, *P *< 0.001). In TBAD patients, serum CDC42 was negatively correlated with Th17 cells (*P *= 0.001), but not Th1 (*P *= 0.130) or Th2 cells (*P *= 0.098). Seven (5.5%) patients experienced in-hospital mortality. Serum CDC42 was reduced in patients who experienced in-hospital mortality vs. those who did not (median (IQR): 191.0 (145.0–345.0) pg/ml vs. 451.5 (298.3–766.8) pg/ml, *P *= 0.006). By receiver operating characteristic analysis, serum CDC42 showed a good ability for estimating in-hospital mortality [area under curve = 0.809, 95% confidence interval (CI) = 0.662–0.956]. By the multivariate logistic regression analysis, elevated serum CDC42 [odd ratio (OR) = 0.994, 95% CI = 0.998–1.000, *P *= 0.043] was independently correlated with lower risk of in-hospital mortality, while higher age (OR = 1.157, 95% CI = 1.017–1.316, *P *= 0.027) was an independent factor for increased risk of in-hospital mortality.

**Conclusion:**

Serum CDC42 negatively associates with Th17 cells and is independently correlated with decreased in-hospital mortality risk in TBAD patients.

## 1 Introduction

Aortic dissection (AD) is caused by an aortic intimal tear, which facilitates blood flow into the medial layer of the aortic wall, subsequently dividing the original vessel into true and false lumens (1). Stanford type B aortic dissection (TBAD) accounts for nearly one-third of all AD cases, with involvement restricted to the descending aorta, including the descending thoracic and the abdominal aorta (2). The major treatment for TBAD is medical therapy, with the goal of minimizing changes in the aortic wall pressure via controlling blood pressure and heart rate (HR) (3). Nevertheless, some TBAD patients who receive medical therapy will still experience disease progression and develop hypertension, aortic rupture, or malperfusion syndrome, which can lead to death (3–6). Currently, the in-hospital mortality of TBAD patients ranges from 2.9% to 17% (7–10); therefore, it is necessary to find some biomarkers for estimating the risk of in-hospital mortality in these patients need to be identified.

Cell division cycle 42 (CDC42) is a small guanosine triphosphatase (GTPase) of the Rho families that regulates the actin cytoskeleton, cell morphology, proliferation, migration (11). Several studies have shown that CDC42 participates in atherosclerosis and vascular stiffness (12–14). For example, one study elucidates that CDC42 regulates chronic inflammation and plaque formation to participate in atherosclerosis (12). In another study, CDC42 modulates vascular calcification via the protein kinase B signaling pathway to affect vascular stiffness (14, 15). Besides, CDC42 also regulates the release of collagen type I from vascular smooth muscle cells to affect vascular stiffness (13, 16). In addition, CDC42 is reported to be a regulator of CD4^+^ *T* cell differentiation (17–19). For instance, one study discloses that the deficiency of CDC42 enhances the differentiation of naïve *T* cells into T-helper (Th) 1 cells *in vitro* (17). Another study shows that the deletion of CDC42 promotes Th17 differentiation by facilitating glycolysis (19). Given that atherosclerosis, vascular stiffness, and abnormal differentiation of CD4^+^ *T* cells are involved in TBAD progression (20–22), CDC42 is suspected to serve as a biomarker for TBAD patients in clinical practice. However, the role of CDC42 in estimating TBAD risk and in-hospital mortality has not been fully explored, and limited relevant evidence is available thus far.

Hence, to contribute valuable insights to clinical evidence and to assess the potential of CDC42 as a specific biomarker for predicting the short-term survival of TBAD patients in clinical practice, this study analyzed the correlation of serum CDC42 level with Th1, Th2, and Th17 cells, as well as in-hospital mortality in TBAD patients.

## 2 Methods

### 2.1 Patients and healthy subjects

This study consecutively enrolled 127 TBAD patients between May 2019 and March 2023. The inclusion criteria for the patients were as follows: (a) diagnosed with TBAD according to the results of magnetic resonance angiography (MRA) or aortic computed tomography angiography (CTA); (b) aged ≥18 years; and (c) willing to provide peripheral blood. The exclusion criteria were as follows: (a) had a history of cardiac surgery or aortic surgery; (b) had aortic dissection secondary to trauma; (c) had Marfan syndrome (MFS) at the same time; (d) had a serious uncontrolled infection; and (e) had a solid tumor or malignant hematological disease. This study also enrolled 30 healthy individuals (for whom the male-to-female ratio was 2:1) from the same period as healthy controls (HCs). The number of HCs was significantly less than the number of patients due to the difficulty in obtaining informed consent from the HCs and the consideration of cost savings. The inclusion criteria for HCs were as follows: (a) had a normal physical examination result, (b) were aged 45–70 years, and (c) were willing to provide peripheral blood. The exclusion criteria for HCs were based on the exclusion criteria. This study was approved by the Ethics Committee of Xingtai People's Hospital. All the patients and HCs provided written informed consent.

### 2.2 Peripheral blood collection and detection

Peripheral blood was collected within 24 h of the patient's visit. Peripheral blood was also collected from the HCs after enrollment.

A portion of the peripheral blood from the patient was centrifuged, and the serum was separated. CDC42 was detected in the serum using an enzyme-linked immunosorbent assay (ELISA). The kit was purchased from JINGMEI Biotechnology Company (China). The remaining peripheral blood was tested for Th1, Th2, and Th17 cells using flow cytometry (FCM). The following antibodies were used during detection: human anti-CD4-fluorescein isothiocyanate (FITC) Monoclonal Antibody (MoA), human anti-interferon (IFN)-γ-phycoerythrin (PE) MoA, human anti-interleukin (IL)-4-allophycocyanin (APC) MoA, and human anti-IL-17A-FITC MoA. All of the reagents were purchased from Thermo Fisher Company, USA. All the experimental procedures were performed in strict accordance with the instructions.

The peripheral blood from HCs was similarly centrifuged to obtain the serum. CDC42 in the serum was detected via ELISA. The kit was also purchased from JINGMEI Biotechnology Company (China). The experimental procedure was performed strictly following the instructions.

### 2.3 Data collection and outcomes

Baseline characteristics, including age, gender, body mass index (BMI), smoker, hypertension, diabetes, HR, systolic blood pressure (SBP), diastolic blood pressure (DBP), ejection fraction (EF), spiral tear, abdominal vascular involvement, and limb ischemia, were collected from patients after admission. Biochemical indices, including hemoglobin (Hb), white blood count (WBC), platelet (PLT), triglyceride (TG), total cholesterol (TC), low-density lipoprotein cholesterol (LDL-C), D-Dimer, high-density lipoprotein cholesterol (HDL-C), serum creatinine (Scr), and C-reactive protein (CRP), were also collected at the same time. The patients received optimal medication treatment with or without thoracic endovascular aortic repair (TEVAR) based on their conditions. Among the one hundred and twenty-seven patients who received optimal medication treatment, thirty (23.6%) underwent TEVAR.

After the admission of the TBAD patients, the deaths were recorded, and the in-hospital mortality was calculated.

### 2.4 Data analysis

SPSS 22.0 (IBM Corp., USA) was used as a software for analysis. The Kolmogorov–Smirnov test was used to examine the normality of continuous variables. Normally distributed continuous variables were shown using mean ± standard deviation (SD). Skewed distributed continuous variables were shown using median and interquartile range (IQR). Categorical variables were presented using numbers (percentages). Mann–Whitney *U*, Student's t, Chi-square, and Fisher's exact tests were used for the comparative analyses. The receiver operating characteristic (ROC) curve was used to demonstrate the distinguished efficiency of CDC42. Spearman's test was used to evaluate the correlation between two variables. Univariate and step-forward multivariate logistic regression analyses were used to evaluate the associations between factors and in-hospital mortality. *P* < 0.050 was considered to indicate a significant difference.

## 3 Results

### 3.1 Clinical features and biochemical indexes

The mean age of TBAD patients and HCs were 55.7 ± 7.5 years and 57.4 ± 7.0 years, respectively (*P *= 0.245). Ninety (70.9%) TBAD patients and 20 (66.7%) HCs were male (*P *= 0.651). The median (IQR) BMI was 25.2 (22.8–28.3) kg/m^2^ in TBAD patients and 23.6 (21.2–25.4) kg/m^2^ in HCs (*P *= 0.001). A total of 64 (50.4%) TBAD patients and 8 (26.7%) HCs were smokers (*P *= 0.019). In addition, 94 (74.0%) TBAD patients but no HCs were diagnosed as hypertension (*P *< 0.001). HR was 83.0 (IQR: 77.0–92.0) bpm and 75.5 (69.8–86.0) bmp in TBAD patients and HCs, respectively (*P *< 0.001). Regarding blood pressure, the mean SBP was 156.0 ± 13.3 mmHg in TBAD patients and 107.9 ± 8.6 mmHg in HCs (*P *< 0.001). The mean DBP was 88.7 ± 10.8 mmHg and 76.3 ± 8.4 mmHg in TBAD patients and HCs, correspondingly (*P *< 0.001). The median (IQR) EF was 59.0% (55.0%−62.0%). In addition, there were 54 (42.5%) patients with spiral tears, 47 (37.0%) patients with abdominal vascular involvement, and 9 (7.1%) patients with limb ischemia. The detailed features of TBAD patients and HCs were listed in Table 1.

**Table 1:** Baseline characteristics of TBAD patients and HCs.

Items	TBAD (*N* = 127)	HCs (*N* = 30)	*P* value
Age (years), mean ± SD	55.7 ± 7.5	57.4 ± 7.0	0.245
Male, *n* (%)	90 (70.9)	20 (66.7)	0.651
BMI (kg/m^2^), median (IQR)	25.2 (22.8–28.3)	23.6 (21.2–25.4)	0.001
Smoker, *n* (%)	64 (50.4)	8 (26.7)	0.019
Hypertension, *n* (%)	94 (74.0)	0 (0.0)	<0.001
Diabetes, *n* (%)	15 (11.8)	0 (0.0)	0.077
HR (bpm), median (IQR)	83.0 (77.0–92.0)	75.5 (69.8–86.0)	<0.001
SBP (mmHg), mean ± SD	156.0 ± 13.3	107.9 ± 8.6	<0.001
DBP (mmHg), mean ± SD	88.7 ± 10.8	76.3 ± 8.4	<0.001
EF (%), median (IQR)	59.0 (55.0–62.0)	–	–
Spiral tear, *n* (%)	54 (42.5)	–	–
Abdominal vascular involvement, *n* (%)	47 (37.0)	–	–
Limb ischemia, *n* (%)	9 (7.1)	–	–
Optimal medication treatment, *n* (%)	127 (100.0)	–	–
TEVAR, *n* (%)	30 (23.6)	–	–

TBAD, Stanford type B aortic dissection; SD, standard deviation; BMI, body mass index; IQR, inter-quartile range; HR, heart rate; SBP, systolic blood pressure; DBP, diastolic blood pressure; EF, ejection fraction; TEVAR, thoracic endovascular aortic repair.

The median (IQR) proportions of Th1, Th2, and Th17 cells were 16.7% (13.8%−20.9%), 10.2% (8.1%−11.9%), and 4.4% (3.4%−6.0%), respectively. The specific baseline biochemical indexes of TBAD patients were shown in Table 2.

**Table 2:** Baseline biochemical indexes of TBAD patients.

Items	TBAD (*N* = 127)
Hb (g/L), median (IQR)	130.0 (122.0–138.0)
WBC (10^9^/L), median (IQR)	9.0 (6.4–12.8)
PLT (10^9^/L), median (IQR)	200.0 (164.0–244.0)
Scr (μmol/L), median (IQR)	88.3 (80.9–96.4)
TG (mmol/L), median (IQR)	1.4 (0.7–1.8)
TC (mmol/L), median (IQR)	4.3 (3.5–5.3)
LDL-C (mmol/L), median (IQR)	2.6 (2.1–3.6)
HDL-C (mmol/L), median (IQR)	1.1 (0.9–1.2)
D-Dimer (μg/ml), median (IQR)	3.9 (2.3–13.8)
CRP (mg/L), median (IQR)	22.6 (13.6–37.3)
Th1 (%), median (IQR)	16.7 (13.8–20.9)
Th2 (%), median (IQR)	10.2 (8.1–11.9)
Th17 (%), median (IQR)	4.4 (3.4–6.0)

TBAD, Stanford type B aortic dissection; Hb, hemoglobin; IQR, interquartile range; WBC, white blood count; PLT, platelet; Scr, serum creatinine; TG, triglyceride; TC, total cholesterol; LDL-C, low-density lipoprotein cholesterol; HDL-C, high-density lipoprotein cholesterol; CRP, C-reactive protein; Th1, T-helper 1; Th2, T-helper 2; Th17, T-helper 17.

### 3.2 Serum CDC42

**Figure 1:**
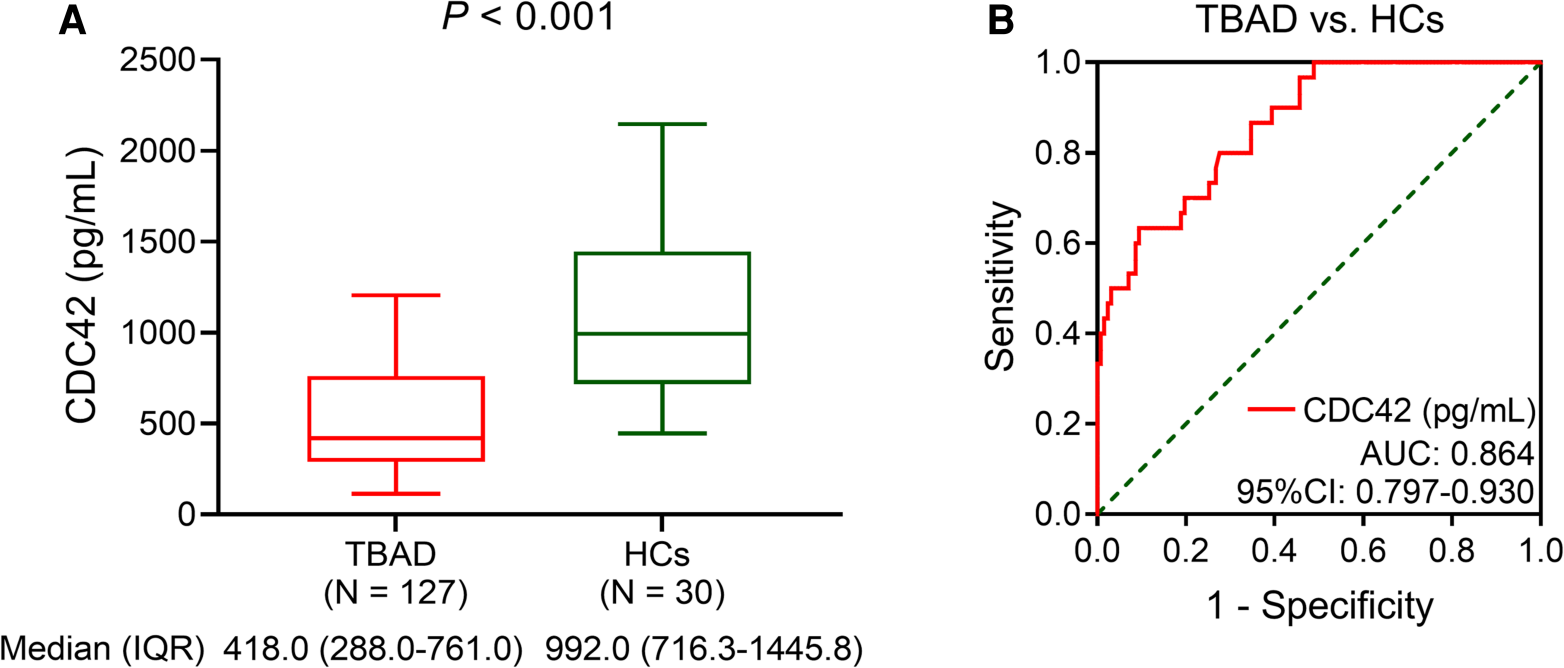
Serum CDC42 was decreased in TBAD patients vs. HCs. Comparison of serum CDC42 level between TBAD patients and HCs (**A**) and its relevant ROC curve (**B**).

Serum CDC42 was decreased in TBAD patients vs. HCs (*P *< 0.001). In detail, the median serum CDC42 was 418.0 (IQR: 228.0–761.0) pg/ml in TBAD patients and 992.0 (IQR: 716.3–1,445.8) pg/ml in HCs (Figure 1A). Serum CDC42 exhibited a good value for distinguishing TBAD patients from HCs (area under the curve (AUC): 0.864, 95% confidence interval (CI): 0.797–0.930, Figure 1B).

In addition, serum CDC42 was not varied between TBAD patients with and without TEVAR (*P *= 0.241) (Supplementary Figure S1).

### 3.3 Correlation of serum CDC42 with Th1, Th2, Th17 cells and in-hospital mortality in TBAD patients

**Figure 2:**
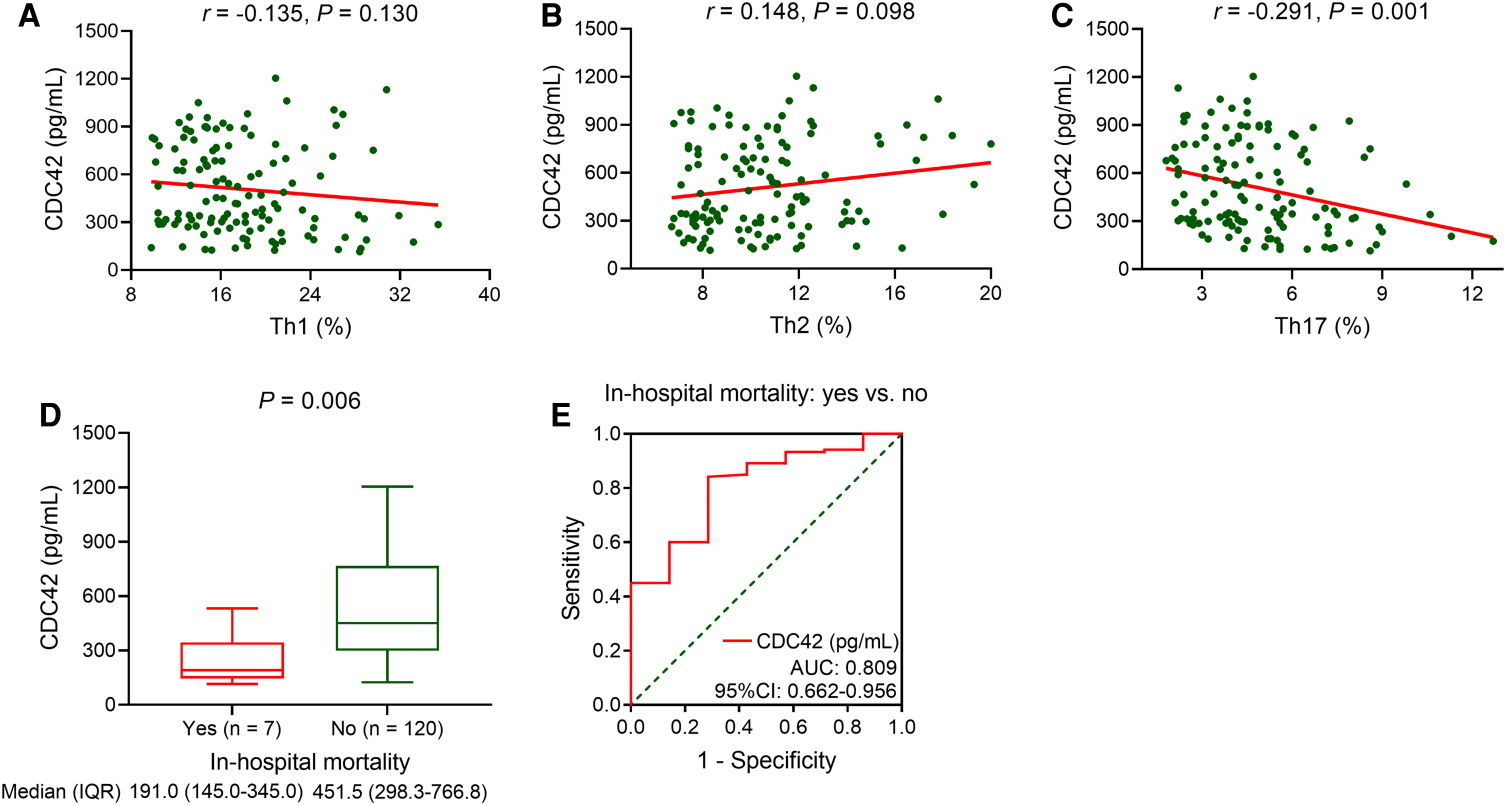
Serum CDC42 was reversely linked with Th17 cells and in-hospital mortality in TBAD patients. Linkage of serum CDC42 with Th1 (**A**), Th2 (**B**), and Th17 (**C**) cells in TBAD patients. Linkage of serum CDC42 with in-hospital mortality in TBAD patients (**D**), and its relevant ROC curve (**E**).

Serum CDC42 was not associated with Th1 (*P *= 0.130, Figure 2A) or Th2 cells (*P *= 0.098, Figure 2B) in TBAD patients, but it was inversely linked with Th17 cells (*P *= 0.001, Figure 2C). Serum CDC42 was negatively correlated with in-hospital mortality in TBAD patients (*P *= 0.006). The median (IQR) serum CDC42 was 191.0 (145.0–345.0) pg/ml and 451.5 (298.3–766.8) pg/ml in patients who experienced in-hospital mortality and those who did not, respectively (Figure 2D). Serum CDC42 showed a good ability to estimate in-hospital mortality in TBAD patients (AUC: 0.809, 95% CI: 0.662–0.956, Figure 2E).

### 3.4 Univariate logistic regression analysis for in-hospital mortality

Among the TBAD patients, age (*P *= 0.014) and SBP (*P *= 0.011) were positively linked with in-hospital mortality. On the contrary, male (*P *= 0.025) was related to decreased in-hospital mortality. The other baseline characteristics were not linked with in-hospital mortality (all *P *> 0.050, Table 3).

**Table 3:** Univariate logistic regression analysis on baseline characteristics of in-hospital mortality in TBAD patients.

Items	In-hospital mortality		OR	95% CI	*P* value
	Yes (*n* = 7)	No (*n* = 120)			
Age (years), mean ± SD	63.0 ± 6.2	55.3 ± 7.4	1.151	1.029–1.287	0.014
Male, *n* (%)	2 (28.6)	88 (73.3)	0.145	0.027–0.787	0.025
BMI (kg/m^2^), median (IQR)	26.4 (22.4–32.4)	25.0 (22.8–28.3)	1.072	0.876–1.312	0.500
Smoker, *n* (%)	3 (42.9)	60 (50.0)	1.333	0.286–6.214	0.714
Hypertension, *n* (%)	6 (85.7)	88 (73.3)	2.182	0.253–18.830	0.478
Diabetes, *n* (%)	2 (28.6)	13 (10.8)	3.292	0.579–18.719	0.179
HR (bpm), median (IQR)	90.0 (80.0–114.0)	83.0 (77.0–91.8)	1.043	0.996–1.092	0.075
SBP (mmHg), mean ± SD	170.0 ± 11.4	155.2 ± 13.0	1.107	1.024–1.197	0.011
DBP (mmHg), mean ± SD	92.4 ± 8.0	88.5 ± 11.0	1.036	0.962–1.116	0.351
EF (%), median (IQR)	58.0 (46.0–66.0)	59.0 (55.0–62.0)	0.956	0.860–1.063	0.407
Spiral tear, *n* (%)	2 (28.6)	52 (43.3)	0.523	0.098–2.804	0.449
Abdominal vascular involvement, *n* (%)	4 (57.1)	43 (35.8)	2.388	0.510–11.168	0.269
Limb ischemia, *n* (%)	1 (14.3)	8 (6.7)	2.333	0.250–21.813	0.458

TBAD, Stanford type B aortic dissection; OR, odd ratio; CI, confidence intervals; SD, standard deviation; BMI, body mass index; HR, heart rate; IQR, interquartile range; SBP, systolic blood pressure; DBP, diastolic blood pressure; EF, ejection fraction.

Besides, serum CDC42 (*P *= 0.034) was negatively related to in-hospital mortality in TBAD patients. However, Scr (*P *= 0.021), CRP (*P *= 0.010), and Th17 cells (*P *= 0.025) were positively associated with in-hospital mortality. But the other baseline biochemical indexes were not related to in-hospital mortality (all *P *> 0.050, Table 4).

**Table 4:** Univariate logistic regression analysis on baseline biochemical indexes of in-hospital mortality in TBAD patients.

Items	In-hospital mortality		OR	95% CI	*P* value
	Yes (*n* = 7)	No (*n* = 120)			
CDC42 (pg/ml), median (IQR)	191.0 (145.0–345.0)	451.5 (298.3–766.8)	0.993	0.987–0.999	0.034
Hb (g/L), median (IQR)	125.0 (117.0–132.0)	130.0 (123.0–138.0)	0.961	0.901–1.024	0.220
WBC (10^9^/L), median (IQR)	10.6 (8.7–15.3)	8.7 (6.4–12.8)	1.093	0.957–1.247	0.189
PLT (10^9^/L), median (IQR)	223.0 (163.0–244.0)	197.0 (164.3–245.5)	1.004	0.991–1.017	0.579
Scr (μmol/L), median (IQR)	100.3 (91.9–113.0)	87.6 (79.7–95.0)	1.046	1.007–1.087	0.021
TG (mmol/L), median (IQR)	1.6 (1.2–2.6)	1.4 (0.7–1.8)	1.763	0.611–5.093	0.295
TC (mmol/L), median (IQR)	5.1 (3.6–5.5)	4.2 (3.5–5.3)	1.362	0.724–2.561	0.338
LDL-C (mmol/L), median (IQR)	3.7 (2.1–3.8)	2.6 (2.1–3.5)	1.542	0.804–2.959	0.193
HDL-C (mmol/L), median (IQR)	1.0 (0.8–1.1)	1.1 (0.9–1.2)	0.097	0.003–3.208	0.191
D-Dimer (μg/ml), median (IQR)	18.2 (3.7–20.8)	3.8 (2.2–12.9)	1.057	0.992–1.128	0.088
CRP (mg/L), median (IQR)	49.4 (35.6–70.5)	21.9 (13.5–36.0)	1.033	1.008–1.058	0.010
Th1 (%), median (IQR)	18.6 (13.3–28.3)	16.5 (13.9–20.9)	1.067	0.944–1.206	0.301
Th2 (%), median (IQR)	9.5 (7.2–11.1)	10.3 (8.1–12.1)	0.834	0.593–1.173	0.297
Th17 (%), median (IQR)	6.0 (5.3–8.6)	4.4 (3.2–5.7)	1.416	1.045–1.920	0.025

TBAD, Stanford type B aortic dissection; OR, odd ratio; CI, confidence intervals; CDC42, cell division cycle 42; IQR, interquartile range; Hb, hemoglobin; WBC, white blood count; PLT, platelet; Scr, serum creatinine; TG, triglyceride; TC, total cholesterol; LDL-C, low-density lipoprotein cholesterol; HDL-C, high-density lipoprotein cholesterol; CRP, C-reactive protein; Th1, T-helper 1; Th2, T-helper 2; Th17, T-helper 17.

### 3.5 Multivariate logistic regression analysis for in-hospital mortality

In TBAD patients, elevated serum CDC42 [odd ratio (OR): 0.994, 95% CI: 0.998–1.000, *P *= 0.043] was independently correlated with decreased in-hospital mortality. Inversely, higher age (OR: 1.157, 95% CI: 1.017–1.316, *P *= 0.027) was an independent factor for increased in-hospital mortality (Table 5).

**Table 5:** Step-forward multivariate logistic regression analysis of in-hospital mortality in TBAD patients.

Items	OR	95%CI	*P* value
CDC42 (pg/ml)	0.994	0.998–1.000	0.043
Age (years)	1.157	1.017–1.316	0.027

TBAD, Stanford type B aortic dissection; OR, odd ratio; CI, confidence intervals; CDC42, cell division cycle 42.

## 4 Discussion

The main findings of the current study were as follows: First, CDC42 was reduced in TBAD patients vs. HCs, with a good ability to estimate TBAD risk. Second, CDC42 was negatively linked with Th17 cells in TBAD patients. Third, CDC42 was declined in TBAD patients with in-hospital mortality vs. those without. Fourth, CDC42 was independently, negatively associated with in-hospital mortality in TBAD patients.

Based on the evidence that CDC42 plays a critical role in regulating atherosclerosis and vascular stiffness (20, 22), some studies have investigated the abnormal level of CDC42 in patients with cerebrocardiovascular disease (23, 24). For example, in one previous study, serum CDC42 is reduced in stroke patients compared to healthy individuals (23). Another study reveals that serum CDC42 is decreased in cardiovascular disease patients compared to the controls (24). Similarly, this study found that serum CDC42 was reduced in TBAD patients vs. HCs. The possible reasons could be as follows: (1) Reduced CDC42 could increase vascular stiffness and decline vascular elasticity by suppressing actomyosin contractility, leading to an increased risk of aortic tearing (25, 26). (2) Decreased CDC42 could increase inflammatory cell infiltration to impair vessel vascular integrity, especially Th17 cells, which secreted interleukin-22 to exacerbate the progression of AD (27–29). Therefore, serum CDC42 was decreased in TBAD patients than HCs. Additionally, the ROC analysis suggested that serum CDC42 showed a good ability to discriminate TBAD patients from HCs. Considering that ELISA was wildly used in laboratory examinations and that its detection time was relatively short, the use of the serum CDC42 to differentiate TBAD patients from HCs was easily accessible in clinical practice. Moreover, a cost-effective investigation of serum CDC42 detection for TBAD diagnosis was meaningful for future clinical practice.

In addition to the abnormal level of CDC42, its linkage with Th17 cells has been indicated in cerebrocardiovascular disease by several clinical studies (30, 31). For example, one study discloses that CDC42 negatively associates with Th17 cells in patients with coronary heart disease (30). Another study displays that reduced CDC42 is related to increased Th17 cells in acute ischemic stroke patients (31). In this study, the findings showed that serum CDC42 was negatively linked with Th17 cells in TBAD patients, which was consistent with the findings of previous studies (30, 31). The probable explanations could be as follows: (1) A reduction in CDC42 promoted glycolysis to enhance Th17 differentiation (19). (2) Decreased CDC42 inactivated Toll-like receptors to promote Th17 cell differentiation (32, 33). (3) A reduction in CDC42 can increase the level of interleukin-1β, and the latter facilitated the differentiation of CD4^+^ *T* cells into Th17 cells (34, 35).

Interestingly, the TBAD patients in the present study had a slightly higher BMI, but their lipid panel is basically in the normal range. The probable explanation could be that TBAD patients often received pharmacological treatments for managing metabolic diseases, which also exerted the efficacy in controlling lipid levels. Hence, the lipid profile of TBAD patients in this study was basically within the normal range, but their BMI was elevated.

Notably, this study revealed that serum CDC42 was declined in TBAD patients who experienced in-hospital mortality versus those who did not, and it showed an independent, negative association with in-hospital mortality in TBAD patients. The possible explanations could be as follows: (1) Reduced CDC42 aggravated vascular stiffness, suggesting the increased severity of TBAD (26), which along with the increased in-hospital mortality risk (36). (2) Decreased CDC42 expression increased neutrophil motility, which increased the risk of bleeding and death (37, 38). (3) A decreased CDC42 level was linked to an increase in Th17 cells in the present study, and the latter was associated with mortality in patients with AD (39). Hence, serum CDC42 exhibited an independent, negative correlation with in-hospital mortality in TBAD patients.

According to one previous study, Th17 cells, but not Th1 or Th2 cells, are related to 30-day mortality in Stanford type A AD patients (39). In this study, univariate logistic regression analysis revealed that Th17 cells were linked to an increased risk of in-hospital mortality in TBAD patients, which was similar to the findings of previous study (39). Besides, there are several traditional cardiovascular factors associated with mortality in TBAD patients, such as age, hypertension, diabetes, and smoking (4, 44, 45). For example, the vascular function of hypertension patients is impaired, vascular stiffness is aggravated, and the latter is related to cardiovascular mortality (22, 46). In addition, smoking is associated with elevated oxidative stress and proinflammatory factors in the aorta, which are associated with the progression of TBAD (47–49). The current study also found that higher age was an independent factor for increased in-hospital mortality in TBAD patients, which was similar to the findings of one previous study (40). However, other traditional cardiovascular factors were not linked with in-hospital mortality in this study. The possible reason could be the small number of endpoint events, which weakened the statistical power.

Some limitations existed in this study. To begin with, this study enrolled TBAD patients who received conservative treatment. Thus, the role of CDC42 in post-surgery TBAD patients needed further exploration. Second, given that the relevant studies are few so far, and the death events of the current study were relatively few, the evidence for specificity of serum CDC42 to estimate in-hospital mortality in TBAD patients remained limited, which needed more investigations for validation. Third, despite that the lower in-hospital mortality in males (vs. females) was similar to previous studies (41–43), the population of patients and HCs in this study were predominantly male, which might cause selective bias. Thereby investigations with a more diverse and representative sample were warranted to validate the current findings. Fourth, this study did not detect the kinetic of CDC42, which could be further investigated. Finally, the current study explored the role of CDC42 in TBAD patients during hospitalization, but its ability to predict disease progression in patients after discharge in the long-term was unclear.

In summary, serum CDC42 negatively associates with Th17 cells and has an independent, negative correlation with in-hospital mortality in TBAD patients. These findings may provide evidence for identifying TBAD patients at a high risk of in-hospital mortality by detecting serum CDC42 and conduce to more individualized management. However, further large-scale studies with longer follow-up durations are needed to validate these findings.

## Data Availability

The original contributions presented in the study are included in the article/Supplementary Material, further inquiries can be directed to the corresponding author.

## Supplementary material

Supplementary Figure S1 Serum CDC42 was not varied between TBAD patients with and without TEVAR.
