# Supplementary figures and images for: Correlation of the serum cell division cycle 42 with CD4+ T cell subsets and in-hospital mortality in Stanford type B aortic dissection patients
Source: Front Cardiovasc Med. 2024 Feb 27;11:1324345. doi: 10.3389/fcvm.2024.1324345 (PMC10927740; doi:10.3389/fcvm.2024.1324345)

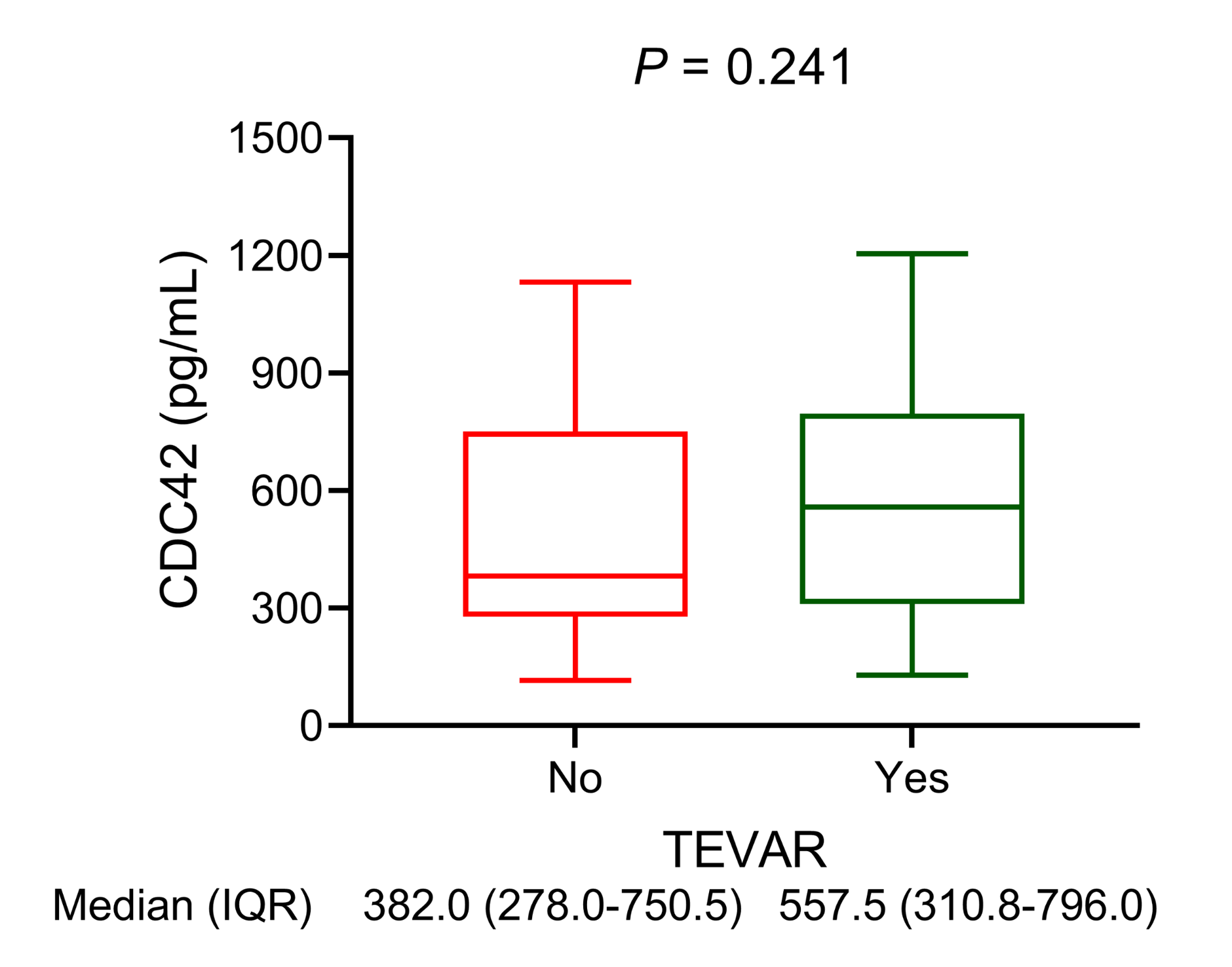

Supplement: Supplementary Figure S1 — Serum CDC42 was not varied between TBAD patients with and without TEVAR. [file Image1.tif]
